# Integrating harm reduction interventions for older adults in opioid treatment programs: a qualitative study of patient and provider perspectives

**DOI:** 10.1186/s12954-026-01503-0

**Published:** 2026-07-08

**Authors:** Wayne Kepner, Benjamin H. Han, Annie L. Nguyen, Raúl Bejarano-Romero, Nora Satybaldiyeva, Alison A. Moore, Angela R. Bazzi

**Affiliations:** 1 Department of Medicine, School of Medicine, University of California San Diego, 9500 Gilman Drive, San Diego, CA 92093 USA; 2 School of Social Work, San Diego State University, 5500 Campanile Drive, San Diego, CA 92093 USA; 3 Stein Institute for Research on Aging, University of California San Diego, 9575 Gilman Dr, La Jolla, CA 92093 USA; 4 Herbert Wertheim School of Public Health, University of California San Diego, 9500 Gilman Drive, La Jolla, CA 92093 USA; 5 Department of Community Health Sciences, Boston University School of Public Health, 801 Massachusetts Avenue, Boston, MA 02118 USA

**Keywords:** Aaging, Oopioid use disorder, Mmethadone, Harm reduction, Implementation

## Abstract

**Background:**

The prevalence of opioid use disorder (OUD) among older adults has increased rapidly, yet this population faces unique challenges in accessing harm reduction services within opioid treatment programs (OTPs). The objective of this study was to explore patient and provider perspectives on the acceptability and feasibility of delivering harm reduction interventions to older adults within OTPs.

**Methods:**

From June 2023 to February 2024, we conducted a qualitative study with two OTPs in San Diego County, California. Participants included 25 patients aged 55 and older receiving medications for OUD, and 15 OTP providers. Semi-structured interviews and thematic analysis focused exploring participants’ beliefs, attitudes, and experiences with harm reduction interventions.

**Results:**

Both patients and providers expressed broad acceptability and perceived need for harm reduction services within OTPs. Key barriers identified included intersectional stigma related to age and substance use, lack of age-appropriate interventions, transportation challenges, and technological barriers. Strong patient-provider relationships were viewed as critical for successful implementation; patients emphasized the value of providers with lived experience. Providers highlighted implementation challenges, including resource constraints, sustainability concerns, and the need for integrated care models addressing complex medical and social needs. While providers viewed the frequent contact inherent in OTP settings as an opportunity for relationship building and service delivery, cognitive changes among some patients and multiple chronic conditions could complicate care provision.

**Conclusion:**

Patients and providers report high acceptability for integrating harm reduction services within OTPs tailored to older adults, but significant barriers exist. Addressing these barriers through tailored interventions, improved accessibility, and efforts to reduce stigma may improve care for this vulnerable population. Implementation strategies should focus on establishing sustainable funding mechanisms, strong community partnerships, and ongoing provider training. Finally, harm reduction services may require regular review to ensure they accommodate the unique needs of older adults with OUD.

## Introduction

 In the United States, opioid use disorder (OUD) and opioid-related harms have increased substantially among middle-aged and older adults. Opioid-related deaths among Americans aged 55 and older increased nearly 10-fold between 1999 and 2019 [1]. Among fee-for-service Medicare beneficiaries aged 65 and older, claims-identified OUD prevalence more than doubled between 2013 and 2018 [2]. Fatal drug overdose mortality among adults aged 65 and older also rose sharply between 2002 and 2021, with particularly high rates among non-Hispanic African American older adults [3]. OUD among older adults carries additional implications for public health and clinical practice, as older adults are also entering OUD treatment at increasing rates [4]; however, older adults infrequently access other harm reduction services, presenting a barrier to healthy aging [5]. Despite these concerning trends, there remains limited research on effective strategies to engage older adults in harm reduction services and address their unique needs in OUD treatment programs.

Harm reduction interventions aim to reduce the adverse consequences of substance use and can be particularly beneficial for older adults experiencing increased vulnerability for overdose due to physiological changes and an increase in chronic diseases [6, 7]. Examples of evidence-based harm reduction interventions include overdose education and naloxone distribution, provision of sterile drug use supplies, HIV and HCV testing, and drug checking services [8, 9]. In addition to directly reducing health harms of substance use, these interventions can also support the uptake of other healthcare services that are critical for older adults due to concurrent medication use, comorbidity, and declining physical and cognitive function [10]. Age-appropriate strategies include education on polysubstance use risk and reduced opioid metabolism in older adults [5].

Opioid treatment programs (OTPs) are federally regulated treatment facilities that provide medications for opioid use disorder (MOUD) such as methadone, buprenorphine, and occasionally naltrexone [11]. OTPs typically employ multidisciplinary teams of physicians, nurses, counselors, case managers, and administrative staff to deliver comprehensive care to patients with OUD [11]. However, the extent to which these programs deliver age-appropriate harm reduction interventions to older adults is unclear.

Older adults face a variety of health-related challenges, such as transportation challenges, intersectional stigma, and financial difficulties, which all serve as barriers to accessing harm reduction interventions and addiction treatment [11]. Due to aging-related physiological changes, increased prevalence and severity of chronic diseases, and potentially high numbers of medications used in this population, older adults are particularly vulnerable to the harmful health consequences of drug use [12]. Yet, older adults are often overlooked in harm reduction efforts due to a lack of understanding of the types of services that meet their needs and preferences [13]. Also, ageism among providers—for example, misconceptions that older adults do not use drugs—might lead to underestimation of the needs of older adult patients [14]. Additionally, the stigma associated with being an older adult who has an OUD may prevent the patient from feeling comfortable reaching out for help [15]. Age-related changes such as mobility limitations and memory decline can also serve as a barrier to engaging with traditional harm reduction strategies that may have been designed for younger populations [16, 17]. For example, services in high-traffic areas may be inaccessible to older adults with limited mobility and transportation limitations [18]. The lack of age-appropriate strategies (e.g. access, trained professionals) within addiction care in general, and OTPs specifically, may serve as an additional layer of barriers faced by older adults. There is currently a significant gap in understanding the specific needs and preferences of older adults regarding harm reduction within OTPs. Therefore, this study aims to explore the acceptability and feasibility of delivering harm reduction interventions to older adults within OTPs from the perspectives of socio-demographically diverse older adult patients and OTP staff.

## Methods

### Study design and sample

We partnered with San Diego County’s Behavioral Health Services to identify potential study sites, presenting our planned research to directors of registered Opioid Treatment Programs (OTPs). From eight OTP directors who agreed, we selected two sites that ensured diverse patient demographics. Purposive site selection prioritized geographic and demographic diversity within San Diego County. One site served a predominantly suburban patient population, while the second was located in a community adjacent to the US-Mexico border. Within these sites, we recruited two participant groups: [1] patients and [2] healthcare staff. Patients were eligible if they were at least 55 years of age, actively received treatment with medications for opioid use disorder (MOUD) at the OTPs, spoke English, and could provide written informed consent. The 55 and older age threshold was selected in alignment with substance use literature that recognizes accelerated physiological aging among people with long-term opioid use disorder. Chronic substance use is associated with earlier onset of age-related health conditions, including chronic pain, cognitive changes, and multimorbidity, such that individuals with OUD at age 55 may present with functional profiles more consistent with the general population at 65 or older [13]. We recruited patients through IRB-approved flyers, staff referrals, and waiting area announcements. Healthcare staff participants included both clinical providers (e.g., nurses) and support staff (e.g., case managers, counselors). We employed purposive sampling to capture diverse staff roles and employment durations, ensuring comprehensive perspectives on older adult OTP care. Participants were compensated $50 for their time and participation. Sample size was determined by thematic saturation, defined as the point at which no substantively new themes emerged across successive interviews [19]. Data collection was conducted from June 2023 to February 2024.

### Data collection

Interview questions focused on the acceptability, and perceived challenges to accessing harm reduction interventions for older adults (e.g., HIV prevention, infectious disease testing, naloxone distribution and overdose education, sexual health, safe injection supplies). For all participants, we conducted semi-structured interviews in private rooms within the OTPs to ensure confidentiality and participant comfort. Each interview lasted approximately 45 min and was conducted by trained research staff experienced in qualitative methods and sensitive to the unique challenges faced by older adults with OUD. The principal investigator (B.H.H.) is a board-certified geriatric and addiction medicine physician and conducted the interviews with a trained research associate (W.E.K.). None of the patients were under the clinical care of the principal investigator.

Interview guides were developed iteratively with input from the research team, including investigators with lived experience in harm reduction settings. Questions used person-first language and a narrative framing (e.g., “Can you share your personal journey with opioids?”) to minimize stigma. Provider guide questions were similarly structured to locate challenges in systemic and structural factors rather than individual behavior (e.g., “What barriers exist within your program to delivering harm reduction services to older adults?”). Revisions included the addition of person-first alternatives and updating demographic items to use gender-inclusive language. For patients, questions delved into the acceptability and perceived challenges to accessing these services, while staff and providers were asked about the feasibility and appropriateness of delivering harm reduction programs within OTPs for older adults. The study was guided by an intersectionality framework, which examines how overlapping social identities and structural inequities (e.g., age, substance use history, and socioeconomic status) interact to shape health experiences and access to care [20]. This comprehensive approach aimed to understand both patient experiences and provider perspectives on integrating these vital services. After the first interview for each group of participants, B.H.H. and W.E.K. reviewed the transcripts, debriefed the information, and adjusted the ordering of questions and added probes for subsequent interviews. This iterative process continued for the first 5 interviews of each group, allowing the study team to better address the study objectives by adapting to emerging responses [19]; interviews were audio-recorded with participants’ consent and transcribed verbatim for analysis.

### Data analysis

Interview transcripts were imported into NVivo version 14 [21]. A team-based approach was employed for codebook development [22]. The coding team consisted of three trained research associates (W.E.K., N.S., R.R.B) who developed a codebook through an iterative process of independent open coding, team meetings, and codebook refinement [19]. We employed a reflexive thematic analysis approach that emphasizes iterative engagement with data and team-based interpretation to develop themes that capture meaningful patterns across participant experiences [23]. Two pairs of research associates (W.E.K., N.S. / W.E.K, R.R.B) coded the same transcripts and met regularly to compare code application, discuss emergent findings, resolve discrepancies through consensus, and refine the preliminary codebook until the coding structure was stable. Using the final codebook, one analyst (W.E.K.) independently coded the remaining interview transcripts. Finally, our thematic analysis involved synthesizing coded data to identify key themes through regular consultations across our multidisciplinary team (e.g., epidemiologist, addiction medicine physician, gerontologist) to help ensure a variety of viewpoints for purposes of reflexivity [22].

## Results

### Sample characteristics

Among 25 OTP patient participants, the mean age was 60.5 years (SD = 5; range 55–75), 56% identified as male (Table 1). Drugs commonly used in the past month included heroin and/or fentanyl (44%), methamphetamine (28%), and non-prescribed prescription opioids (8%). Nearly one quarter (23%) reported injection drug use in the last 6 months. Most (94%) who injected reported using heroin, fentanyl, or another synthetic opioid. 17% reported infections related to substance use in the past year. Among 15 program provider participants, our sample consisted of registered nurses (*n* = 4), counselors (*n* = 7), administrative staff (*n* = 2), a clinic director (*n* = 1), and a case manager (*n* = 1). Mean years of employment duration for OTP staff was 8.2 years (SD = 5.4).

**Table 1:** Demographic and chronic disease characteristics of patient participants (*N* = 25)

Characteristic	No. (%)
Age, mean (SD), years	60.5 (5.0)
Age range, years	55–75
*Gender identity*	
Man	14 (56.0)
Woman	10 (40.0)
Transgender	1 (4.0)
*Race/ethnicity*	
Hispanic	14 (56.0)
Non-Hispanic white	8 (32.0)
Non-Hispanic other	3 (12.0)
*Health insurance*	
Medicaid only	20 (80.0)
Medicare/Medicaid	4 (16.0)
Other (VHA/Private/None)	1 (4.0)
Housing status	
Housed	18 (72.0)
Unhoused	7 (28.0)
Medication for opioid use disorder	
Methadone	25 (100)
Years since start of current medication treatment, mean (SD)	4.6 (5.0)
*Chronic diseases*	
Active Hepatitis C	11 (44.0)
Depression	12 (48.0)
Anxiety	7 (28.0)
Diabetes	7 (28.0)
Hypertension	7 (28.0)
Coronary artery disease	2 (8.0)
Cancer	2 (8.0)
Cirrhosis	2 (8.0)
Chronic obstructive pulmonary disease	2 (8.0)
Congestive heart failure	1 (4.0)
Asthma	1 (4.0)
2 or more of the above chronic diseases	23 (92.0)

Percentages are based on the total number of participants in the dataset (*N* = 25)

Some participants may have multiple conditions

### Key themes identified

Thematic analysis of 40 interviews (i.e., 25 patients and 15 staff) yielded three overarching themes. First, intersectional stigma and logistical hurdles emerged consistently across both patient and provider interviews as distinct barriers to harm reduction engagement for older adults. Second, trust and lived experience surfaced as critical determinants of acceptability and engagement, with patients and providers both specifically emphasizing the relational element and trust as foundational for harm reduction delivery in OTP settings. Third, systemic constraints and the need for integrated care models reflected providers’ accounts of resource limitations alongside both patients’ and providers’ recognition that OUD care for older adults requires complex and often multidisciplinary service delivery. These themes were identified through iterative team-based coding and consensus across a multidisciplinary research team.

#### Theme 1: Intersectional stigma and logistical hurdles as distinct barriers to harm reduction engagement among older adults in OTPs

Patients and providers reported significant barriers to accessing harm reduction services both within OTPs and in the community. Specific barriers identified included stigma (e.g. law enforcement, healthcare providers), lack of age-appropriate services, and logistical challenges such as transportation. Age-related stigma emerged as a unique barrier:

As one case manager noted, “They’re [older adults] are ashamed to even go [to a harm reduction program]. Even here [at the OTP] sometimes you see the younger people that are here, and they look. It’s like, oh, man, if I’m ever that old and I’m still here…it’s that stigma.”

Providers recognized intersectional stigma as a significant barrier to older adults specifically requesting harm reduction information or services, with one OTP case manager stating, “They’re [older adults] so afraid of the stigma -- and the history and -- of the trauma -- repeated traumatic experiences with other medical providers, medical facilities, what have you.”

Transportation issues emerged as a major obstacle for older adults. Many patients heavily depend on medical transportation services designed for older adults to get to their OTP, which can be especially difficult to access for older adult patients without stable housing and mobility limitations. While younger clients were able to walk, take public transportation or get rides from friends and family, the more isolated older adult clients relied heavily on insurance-provided medical transportation.

A case manager explained, “A medical transportation, they will [only] pick you up at the same place. So, we can’t set standing appointments if you’re going to be moving around and in different areas all the time.”

This issue was worse for older adults without access or the ability to work smartphones, as the provider continued, “These patients need to have a phone so that they can call. So, if they don’t have a phone, and they’re moving around, they miss a week’s worth of dosing, or their appointments for transportation.”

Consequently, lack of consistent transportation was reported as a key barrier to accessing harm reduction services, and the implementation of any programs within OTPs would have to consider these barriers. For example, older adults may have difficulty traveling to the OTP to access harm reduction supplies (e.g., naloxone, fentanyl test strips, etc.). At the same time, since many patients are attending the clinic daily, the convenience of receiving services all at one location could be a facilitator of service uptake.

Technological barriers also hindered access to information about available community-based harm reduction resources.

As one counselor noted, “…they gotta go online to go get their free naloxone, you know?” In San Diego County, syringe service programs have added online modules for patient procurement of harm reduction supplies, including naloxone. This digital divide particularly affected older adults, who often preferred non-digital methods of communication and information gathering.

#### Theme 2: The critical role of trust and lived experience in fostering acceptability and engagement with harm reduction services

Patients reported a high level of acceptability and perceived need for harm reduction services within OTPs due to their lived experience of engaging with many of these services for decades. Older adult patients were able to directly observe the evolution of community programs over the years and had special appreciation for them. Specific services such as naloxone distribution, overdose education, and HIV/HCV testing were viewed as convenient, beneficial and potentially lifesaving. One participant (55-year-old White male) stated, “I’m all for harm reduction…it saves lives in terms of HIV, in terms of overdosing, and it helps people get off the narcotics.”

Older adult patients also expressed enthusiasm for overdose education and naloxone training at the OTP. Naloxone training was viewed as a way to be seen as a “protector” and “useful” to their friends and family, which older adults valued highly. One participant (61-year-old Hispanic male) shared, “Well, nobody else didn’t know what to do. It made me feel good. People looked at me like, you know what they’re doing. I done helped a whole lot of people with Narcan [naloxone]. I know how to use it. I know that.”

Many patients also described long histories of criminal justice involvement that shaped their experiences with infectious disease and harm reduction. One patient recounted contracting hepatitis C while incarcerated: “I was diagnosed with hepatitis C when I was in prison. I carried it for, like, maybe 17 years. I just recently when I’d seen my doctor, she said that she was going to put me a cure for that.” Another described actively managing infection risk during incarceration: “I did a lot of time in prison… all the years I did in prison -- and doing needles -- I was at a high risk of catching hep C -- and I never caught it… I knew I was getting out, so I didn’t want to come out here with anything that was gonna infect anybody else.”

However, the lack of age-appropriate services was also highlighted as a significant barrier which reduced enthusiasm for some services. Patients emphasized the importance of tailored harm reduction education and services that address the unique needs, experiences, and drug use patterns of older adults. The age and experience of providers emerged as key determinants of patient engagement which could serve as both a barrier and a facilitator depending on levels of trust.

A 59-year-old, formerly incarcerated patient commented: “I think that a drug counselor should be a person that has been through that lifestyle. Not a person that just gets it out of the book. Because they don’t know what we’re feeling. They don’t know what we’ve been through or what we feel. They only understand what they read.”

Another patient (67-year-old Hispanic male) echoed this sentiment, stating, “Some of these counselors, they don’t know what they’re talking about. And some do. And the ones that do are the ones that already got out of that junk and using and all that, and they became a counselor. Those are the ones that know where you’re coming from and everything.”

Both patients and providers emphasized the critical role of strong patient-provider relationships in successfully implementing harm reduction interventions for older adults in OTPs. Patients reported that trust was a key factor in their willingness to engage with services.

As one counselor stated, “They just connect really quick --with the ones that open up. They’re like…I think I have syphilis…then I sent them to the neighborhood clinic.” This trust allowed patients to be more open about sensitive health issues.

Providers recognized that their frequent contact with patients facilitated trust-building, which was crucial for engagement. However, social isolation and cognitive changes were noted as barriers to establishing trust because these conditions make it more difficult for providers to establish a relationship.

Peer support emerged as a potential strategy to increase patient-provider trust among older adults. Peer support specialists were viewed as neutral members of the organization who were not compelled to report substance use behaviors to authority figures.

Providers also recognized this need for age-related tailored services to increase engagement. As one counselor explained, peer specialists were people that patients could “relate [to] with no other strings attached.” Patients themselves expressed enthusiasm for peer-led education, with one participant noting, “I think they’d be accepting of it [peer-led interventions]. And they might feel, because of age, they could accept it more…they [older adults] might be more accepting.”

However, a significant barrier to implementing peer support for older adults was the lack of trained peer support specialists. One counselor stated, “I don’t think there’s enough peer support for seniors. Because that’s a relatively new population. So it’s not as established as our youths, or our pregnant, our neo program. This is a new population coming up. So I don’t think enough of it is understood.”

A significant concern among providers was the difficulty of building relationships with and delivering care to older adults showing signs of cognitive decline. As one case manager noted, “I could tell that he had a lot of symptoms of… ‘cause I can’t diagnose dementia, but it seemed like he had a lot of symptoms.” Another provider observed, ‘As you get older you start to develop -- some people have dementia, Alzheimer’s. They might forget that they have an appointment scheduled.’

#### Theme 3: Systemic constraints and the need for integrated care models for older adults in OTPs

Both patients and providers highlighted the importance of comprehensive and individualized care approaches for effectively serving older adults in OTPs. Providers suggested integrating services tailored to the unique needs of older adults and emphasized the value of cultural competence and a philosophy of interdisciplinary care. One nurse explained, “Even if the counselors could call in medical staff, and we can have an intervention together -- I think that that might be helpful. And where it’s more individualized, I think that would be more effective for the elderly population.”

Patients also expressed desire for an integrated approach to care that treated individuals in a holistic manner. One patient (65-year-old African American male) described the challenges of managing multiple chronic conditions: “Cause I got Hep C, you know? So, I have to go see a doctor for my lungs ‘cause I smoke. I got like emphysema. So, I gotta go see a specialist for that. And I have to go see a lung doctor too. A lung doctor and a liver doctor for my Hep C.”

Many providers had a history of attempting to implement a holistic and harm reduction-orientated care approach in their current practice for older adults noting the complex medical needs of the population. For example, one case manager shared, “My goal every time I see an older adult client [is], ‘Do you have a PCP? Who are you with?’ I ask them [this] especially if it is HIV [to] make sure they’re connected, and then once they say who their doctor is, [I ask], ‘Do you know that there’s case management and advocates there that you could speak to?’ ”.

While both patients and providers recognized the importance of comprehensive care, several implementation challenges were identified. Providers noted the complexity of caring for older adults, particularly those with cognitive decline. To address this challenge, a nurse provider suggested involving family members or close friends in the care process: “If they allow bringing in a -- bringing in a family member -- or a close friend. Letting them know, you know, having them come in, ask what we’ve planned. Outline the plan for them so that there’s somebody else that reminds them, ‘Oh, you have an appointment today.”

Resource limitations were also identified as significant barriers to implementing comprehensive care. Providers mentioned issues such as lack of space, funding constraints, and limited staff capacity.

Providers also noted potential challenges to program sustainability: “That’s what has taken away my mobile medical unit. I used to have it every week, but I couldn’t keep the numbers up. So, they were like ”, “Well, it costs too much money to have this whole mobile medical unit here, and a MA, and a driver when you only see three people.” One case manager noted, “The biggest issue [is] space. It’s the office space, you know. Having a designated office for somebody, if you want them to do this job, they’re gonna need an office.”

To address these challenges, providers suggested forming partnerships with community organizations and exploring alternative funding models to ensure sustainability of services.

## Discussion

### Intervention characteristics

#### Age-appropriate interventions

Our findings suggest that to increase uptake, harm reduction interventions for older adults in OTPs should be specifically tailored to address the unique needs and challenges faced by this vulnerable population. Tailoring harm reduction is similar to patient-centered care as both prioritize an individual’s unique needs, values, and goals, fostering a collaborative relationship to develop a personalized plan. However, general patient-centered care typically aims to treat or resolve a health condition, while harm reduction focuses on mitigating the risks associated with substance use without necessarily seeking to eliminate use. Current harm reduction programs, both within OTPs and in the community, are generally geared toward younger populations or not tailored to any age group at all [5, 24]. Results suggested that peer support from individuals with lived experience would be highly age-appropriate and effective for educational forums. Many older adults who have recently been released from a long period of incarceration may not be aware of infectious disease treatment advancements (e.g. hepatitis C), harm reduction interventions (e.g. syringe service programs), and changes to the current drug supply (i.e. fentanyl potency, xylazine) [25–27]. Many older adults spent their formative years in a different era of drug policy and therefore may benefit from increased education about the acceptability and utility of prevention, treatment, and harm reduction [28]. Educating older adults, particularly those with a history of incarceration, about these advancements could increase their willingness to seek care within OTPs.

### Service integration

Older adults in OTPs are more likely to have multiple chronic conditions such as cardiovascular disease, diabetes, and chronic obstructive pulmonary disease [16, 29, 30]. They also have higher rates of mental health issues such as depression, post-traumatic stress disorder, and cognitive decline [13]. Older adults are less likely to seek care for SUDs compared to younger adults [31, 32]. Nonetheless, rates of older adults, 55 and older, who sought treatment for OUD increased swiftly from 2004 to 2013 (41.2%) and were even greater between 2013 and 2015 (53.5%) [33]. This mirrors national trends, including in Southern California, that have also seen large increases in adults aged ≥ 55 entering OTPs, especially for the first time and among older people who use heroin. Current addiction treatment models that are fragmented and fail to consider person-centered holistic approaches to OUD care are thus insufficient to meet the current demands of the aging OTP population [34]. Instead, OTPs should offer multiple *integrated* and low-threshold services beyond OUD treatment, such as harm reduction services, primary care, mental health services, and social support services [35]. Implementing comprehensive care models within OTPs may significantly improve treatment outcomes while reducing healthcare system burden and emergency department utilization [36–38].

Continuing education of providers to ensure cultural humility in regard to the cultural values and beliefs of older adults may support the successful implementation of these interventions [39]. Peer support interventions and programs that address older adults’ social isolation can create socially supportive environments that encourage patient engagement and increase uptake of harm reduction services [40]. Building a holistic, multidisciplinary approach that integrates physical health, mental health, and social support services may be important comprehensively addressing the complex needs of older adults with OUD in OTPs.

### OTP setting considerations

#### Resource constraints

Older adults in OTPs often need a higher level of care, but providers face a fragmented healthcare system that makes it difficult to coordinate care for older adult patients [13]. Challenges included limited office space for ancillary services, staff capacity, and program sustainability; these were identified as significant barriers to implementing additional social service programs targeting older adults. The identified constraints are particularly concerning given that aging patients are increasingly living alone, making the lack of integrated social services even more problematic for those struggling with social isolation [41]. Therefore, providing OTPs with resources to deliver additional social and healthcare services may especially benefit this population.

### Sustainability concerns

While participants were generally in favor of integrating harm reduction services for older adults, sustainability was noted as a concern due to funding limitations and concerns about reach and recipient impact [42]. Targeted harm reduction programs were deemed at risk of being discontinued, once established, due to a lack of reach and effectiveness. Providers noted that reaching the older adult population for services is difficult due to various physical challenges which could jeopardize the sustainability of programmatic efforts [6]. Potential solutions to the challenges of resource constraints and sustainability include forming partnerships with community organizations [43]. Program managers in OTPs might consider collaborative funding models involving government institutions, community organizations, and healthcare systems which would create a more stable funding base. Ultimately, addressing both resource constraints and sustainability of harm reduction interventions for older adults will be contingent on a multidisciplinary approach [44].

### External factors

#### Community context

Consistent with previous literature, tension among community members was noted regarding services such as providing sterile injection equipment [45]. However, community factors were also seen as a source of potential strength. Providers reported positive experiences with referral networks to neighborhood clinics and senior centers, particularly for sexually transmitted infection testing and treatment. Collaborations with senior centers, skilled nursing facilities and even public libraries may serve as potential sources of strength for older adult populations [46].

### Transportation and communication systems

Accessibility and transportation emerged as major obstacles for older adult patients in accessing healthcare services [47]. These transportation challenges are particularly acute for older adults trying to access public transportation [18]. Harm reduction interventions housed in community settings may be difficult to access for older adults who are often traveling to multiple health systems in a single week. These external factors suggest the need for innovative solutions that address both community resistance and transportation barriers. While medical transport can serve as a reliable method for some, challenges remain due to inconsistent coverage of services. Future programs should consider integrating a suite of harm reduction interventions within OTPs to mitigate the barrier of logistical difficulties for older adults who are less likely to travel to multiple locations [18].

An important finding was the role of phone access as a prerequisite for transportation-dependent care. Patients without a working phone could not schedule or confirm medical transportation, and missed appointments risked being removed from transportation services entirely, which in turn jeopardized continuity of dosing. This finding suggests that connecting patients to low-cost phone programs could be an important strategy for supporting access to both transportation and harm reduction services. Additionally, while medical insurance may cover transportation to OTPs, it often does not cover transportation to community-based syringe service programs (SSPs), which are not classified as medical appointments. Given that greater distance to SSPs is associated with increased syringe and equipment sharing [48], integrating SSP-style services directly within OTPs could potentially reduce the logistical burden faced by older adults without reliable transportation.

### Individual characteristics

#### Patient factors

The challenges associated with SUD care for older adults with multiple chronic conditions and/or cognitive decline are well documented [28, 49]. Our findings suggest that these challenges are particularly severe in the OTP setting. The reported difficulties of older adult patients managing multiple healthcare appointments and medications align with existing research on the healthcare burden in older adults with multiple chronic conditions [10]. However, adding the dimension of the rigid OTP system makes the burden more difficult due to stringent regulations that require daily or near-daily attendance to receive opioid agonist medications. The increasing rates of polysubstance use among this population highlights the need for more individualized treatment plans [49]. OTP providers must therefore balance the strict regulatory requirements with the complex healthcare needs of older adults [50] to ensure better treatment outcomes.

The emergence of intersectional stigma, social isolation and geriatric conditions as barriers to trust-building adds to the literature by deepening our understanding of relationship-building challenges in OUD treatment for older adults. While prior research on substance use and older adults has emphasized the importance of provider-patient relationships in SUD treatment [51, 52], our findings suggest that age-related factors create unique obstacles in OTP settings that require specialized approaches and additional training. Technological barriers among older adults accessing harm reduction resources through digital platforms parallel issues about the digital divide in health literacy for older adults [53]. Our findings thus contribute to a growing body of SUD literature, suggesting that successful harm reduction interventions for older adults in OTPs must adopt a more comprehensive approach that integrates SUD treatment with geriatric care principles.

### Provider characteristics

Our findings of providers’ integrated care practices validate prior research and add to the literature on geriatric addiction treatment. While these integrated care efforts show promise, the identified biases about older adults’ sexuality highlight persistent challenges in healthcare delivery that merit further attention [54]. Our findings also suggest the need for targeted interventions to address provider assumptions about aging populations [55] and potential new directions for service delivery models within OTPs that may be effective for older adults. Finally, the reported need for cultural humility training and peer support programs for older adults highlights critical gaps in care that warrant future research [56].

### Process considerations

#### Patient-provider relationships

Both patients and providers noted the importance of the patient-provider relationship for successfully implementing harm reduction services. Our findings suggest that the frequent contact built into OTP settings creates unique opportunities for relationship-building with older adults. Patients and providers both reported success in discussing sensitive health concerns once trust was established, which adds to our understanding of how healthcare relationships in OTP settings can aid in holistic care delivery. Collaborations with community partners may be crucial for serving older adults in OTP settings such as senior centers and skilled nursing facilities [11]. The need for increased data collection and evaluation mechanisms highlights the growing importance of evidence-based program development in SUD care. OTPs may enhance the quality of care for older adults through strategic community partnerships and integrated service delivery models.

### Intersectional stigma

The multiple sources of intersectional stigma and ageism identified in our study contribute to an emerging understanding of how various forms of discrimination interact in healthcare settings [20, 57]. While previous research has documented various stigmas related to opioid treatment [58], age-related stigma creates additional barriers for older adults in OTPs. The reported avoidance of seeking out harm reduction services due to feelings of shame suggests that stigma management strategies must address both addiction-related and age-related discrimination [41, 59]. The mitigation of intersectional stigma may be important for improving patient engagement with services in OTPs.

The influence of past negative healthcare experiences on current treatment engagement adds to existing literature on trauma-informed care. Older adults’ accumulated experiences of healthcare discrimination and trauma [60] may require specialized approaches to trust-building and addiction care delivery [15]. The reported concerns about law enforcement interactions regarding harm reduction supplies highlight the complex interplay between healthcare access and criminal justice system involvement [61, 62]. Older adults are a complex population with a diverse set of experiences that warrant specialized care. Providers must recognize and actively address how past traumas and systemic barriers shape older adults’ treatment engagement in OTP settings.

The development of age-specific stigma reduction interventions, including education and intergenerational social support programs, could help address multiple forms of discrimination simultaneously [14]. Furthermore, the role of OTPs as potential safe spaces suggests potential opportunities for developing targeted interventions that take advantage of existing healthcare relationships. The implementation of these new practices and policies could help improve OUD outcomes for older adults in OTPs. Future research should explore how to effectively implement these interventions in diverse settings.

### Limitations

This study has several limitations. First, data were collected from two OTPs in a single county in Southern California, which may limit generalizability to OTP settings in other geographic or regulatory contexts. Second, the qualitative sample was small by design, and findings reflect the experiences of a specific older adult population in San Diego County. Third, all interviews were conducted in English, which may have excluded Spanish-speaking patients, particularly at the border-adjacent site where a predominantly Latino patient population was served. Fourth, the provider sample did not include physicians or nurse practitioners; as such, prescriber perspectives on harm reduction integration are not represented and remain an important direction for future research. Finally, as with all qualitative interview studies, responses may be subject to social desirability bias, particularly when discussing stigmatized behaviors or critiquing clinic staff and services.

## Conclusion

Our study provides critical insights into the integration of harm reduction services in OTPs for older adults. Patients and providers report high acceptability for integrating harm reduction services within OTPs tailored to older adults, but significant barriers exist. Addressing these barriers through tailored interventions, improved accessibility, and efforts to reduce stigma may improve care for this vulnerable population. Implementation considerations suggest the need for multi-level interventions addressing structural and individual barriers and flexible service delivery models that are better able to accommodate varied patient needs. Success will likely depend on establishing sustainable funding mechanisms and strong community partnerships while ensuring ongoing training and support for providers. Regular evaluation and adaptation of services are important considerations to maintain program effectiveness and responsiveness to patient needs. Future research may focus on developing and evaluating age-appropriate harm reduction interventions for older adults in OTPs; particular attention should be paid to implementation strategies that can overcome the identified barriers. This comprehensive approach, combining evidence-based practices with practical implementation considerations, may support care delivery and outcomes for older adults in OTPs.

## Data Availability

The qualitative data generated and analyzed during this study, consisting of interview transcripts, are not publicly available due to the sensitive nature of the information and to ensure the privacy and confidentiality of the study participants. Requests for data can be made to the corresponding author, and will be considered on a case-by-case basis, subject to appropriate ethical and data sharing agreements and institutional review board approval.
